# Harnessing digital technology for COVID-19 response in Uganda: lessons and implications for future public health emergencies

**DOI:** 10.1136/bmjgh-2023-013288

**Published:** 2023-10-04

**Authors:** Rawlance Ndejjo, Steven Ndugwa Kabwama, Alice Namale, Andrew K Tusubira, Irene Wanyana, Susan Kizito, Suzanne N Kiwanuka, Rhoda K Wanyenze

**Affiliations:** 1 Department of Disease Control and Environmental Health, School of Public Health, College of Health Sciences, Makerere University, Kampala, Uganda; 2 Department of Community Health and Behavioural Sciences, School of Public Health, College of Health Sciences, Makerere University, Kampala, Uganda; 3 Department of Epidemiology and Biostatistics, School of Public Health, College of Health Sciences, Makerere University, Kampala, Uganda; 4 Department of Health Policy, Planning and Management, School of Public Health, College of Health Sciences, Makerere University, Kampala, Uganda

**Keywords:** COVID-19, Public Health

## Abstract

COVID-19 was one of the greatest disruptors of the 21st century, causing significant morbidity and mortality globally. Countries around the world adopted digital technologies and innovations to support the containment of the pandemic. This study explored the use of digital technology and barriers to its utilisation in responding to COVID-19 and sustaining essential health services in Uganda to inform response to future public health emergencies in low-resource settings. We reviewed published and grey literature on the use of digital technology in Uganda’s response from March 2020 to April 2021 and conducted interviews with key informants. We thematically synthesised and summarised information on digital technology use as well as related challenges. During the COVID-19 response, digital technology was used in testing, contact tracing and surveillance, risk communication, supportive supervision and training, and maintenance of essential health services. The challenges with technology use were the disparate digital tools and health information systems leading to duplication of effort; limited access and coverage of digital tools, poor data quality; inaccessibility of data and an inability to support data manipulation, analysis and visualisation. Moreover, the inherent inadequate technology support systems such as poor internet and electricity infrastructure in some areas posed challenges of inequity. The harnessing of technology was key in supporting the COVID-19 response in Uganda. However, gaps existed in access, adoption, harmonisation, evaluation, sustainability and scale up of technology options. These issues should be addressed in preparedness efforts to foster technology adoption and application in public health emergencies with a focus on equity.

**Summary box**

- Globally, digital technology was applied in various ways to support response to the COVID-19 pandemic. However, there is limited information on the application of digital technology in sub-Saharan Africa.
- This study highlights how digital technology supported the COVID-19 response in Uganda, the bottlenecks that require attention and its potential use to inform responses to future public health emergencies.
- The study synthesises the key lessons learnt in the application of digital technologies during the COVID-19 response in Uganda.
- The study provides key recommendations for improving the application of digital technologies in future epidemic responses in Uganda and other similar settings.

## Introduction

COVID-19 was one of the greatest disruptors of the 21st century, causing significant morbidity and mortality globally.^1 2^ The unique, fast-spreading and severe nature of COVID-19 challenged traditional surveillance and outbreak investigation across countries.^3 4^ With the need for timely reporting of cases amidst the high infection and hospitalisation rates, tracing and following contacts required innovative, appropriate and cost-effective approaches.^5^

Digitalisation and technology use in healthcare have been dubbed a predictable step in advancing systems resilience as they improve efficiency, monitor quality and revolutionalise services and products. With the COVID-19 pandemic imposing severe restrictions on how people lived normally, several countries inevitably adopted technology and innovations to support the containment of the pandemic.^6–9^ Digital technology was used in epidemiological surveillance,^10 11^ case identification,^12^ interruption of community transmission,^13^ public communications^14^ and clinical care.^15 16^

Uganda registered its first confirmed case of COVID-19 on 21 March 2020, with the first wave of the disease experienced between August 2020 and February 2021 and the second wave from May to November 2021.^17 18^ To meet the demands presented by the unprecedented crisis and support the health system in controlling the pandemic, technology was adopted to support various aspects of the COVID-19 response.^19 20^ However, there has not been systematic documentation of the use of technology in emergency preparedness and response including for COVID-19 in many sub-Saharan African countries. This study explored the use of digital technology and barriers to its utilisation in responding to COVID-19 and sustaining essential health services in Uganda to inform response to future public health emergencies in low-resource settings.

This study was conducted in Uganda, in eastern Africa, with a population of 41.6 million people.^21^ In 2021, the country had a GDP of US$40.5 billion with a per capita expenditure on health of US$17.^22^ Uganda’s internet penetration rate is estimated at 29.1% while 74.0% of the population owns mobile phones.^23^ This qualitative study was descriptive by design and involved a document review and interviews with key informants (KIs). This study was part of a broader project that evaluated the response to COVID-19 in DRC, Ghana, Nigeria, Senegal and Uganda.^24^

We reviewed published and grey literature on technology use in Uganda during the COVID-19 period. We defined digital technology as electronic tools, systems, devices and resources used to generate, store, process and share data in any of the strategic operations of the response to the pandemic. We searched PubMed, Google Scholar and organisation websites including the Ministry of Health (MOH), WHO, UNICEF and USAID for scientific publications, reports, policies and blogs during the peak COVID-19 period of March 2020 and April 2021. We extracted data into a Microsoft Excel template to summarise the technology involved, how it was applied and what aspect of the response it supported, and to identify barriers and challenges arising from its application among other factors.

After the literature review, we conducted interviews with KIs who were involved in the COVID-19 response in Uganda. The KIs were purposively selected and included: national-level policy-makers such as members of the national committee on continuity of essential health services and those who belonged to COVID-19 response pillars such as surveillance, programme managers such as directors/commissioners in charge of health and clinical services or testing and surveillance activities at the regional level, district health officers, and health facility staff. KIs provided perspectives relating to technology use during the COVID-19 response and how well it worked. Interviews were conducted using an interview guide (online data supplement1) with themes such as contact tracing, surveillance, testing and maintenance of essential health services. We conducted interviews with 20 KIs from November 2020 to March 2021 by phone or virtually using Zoom due to COVID-19 restrictions at the time of the study. Interviews were conducted in English by two members of the research team (SNK (male) and AN (female)) both of whom were public health graduates with experience in qualitative research. Two other members supported notetaking during the interviews. The average duration of the interviews was 45 min.

10.1136/bmjgh-2023-013288.supp1 Supplementary data

Literature review data were synthesised to form themes on the scope of available information and highlight gaps which were used to refine the KI interview guide. All KI interviews were audiorecorded and transcribed verbatim. The analysis of transcripts involved the generation of a codebook apriori on which data were deductively coded following the semantic approach and thematically analysed. The presentation of results is supported by quotations from KI interviews and guided by the Consolidated Criteria for Reporting Qualitative Research guidelines.^25^

As this study was interested in the policy-makers’ and health workers’ perspectives, no patients or members of the public were involved in the study design, setting of the research questions, interpretation or writing up of results, or reporting of the research.

## Digital technology use in COVID-19 response in Uganda

During the COVID-19 pandemic, Uganda applied technology to support testing, contact tracing and surveillance, and risk communication and community mobilisation. Technology also supported supportive supervision and training and maintenance of essential health services, as described below and summarised in table 1.

**Table 1:** Use of technology in Uganda’S COVID-19 response

Aspect of COVID-19 response	Application of technology
Testing	Instant access to COVID-19 laboratory results, their dispatch and management to support quick action. Communication of results and follow-up of COVID-19-positive clients. Registration and payment of testing fees such as at the airport. Verification of results and traceability to the laboratory test centre. Reporting of testing data by facilities. A mobile phone application supported the issuance of COVID-19 digital certificates.
Contact tracing and surveillance	Tracing of contacts. Transmission/sharing of data such as daily situational reports from the lower levels to the centre or within the same levels. Conduct surveillance such as using the Electronic Integrated Disease Surveillance and Response System. Screening, clearance and tracking of travellers at border districts. Website with dashboard providing updates on surveillance.
Risk communication and community mobilisation	Develop multilingual information and communication campaigns. Share COVID-19 information and messages through mobile phones and social media. Citizen engagement for feedback and data collection on important COVID-19 impacts. Obtain and monitor incidents, beliefs and opinions about the pandemic.
Supportive supervision and training	Supportive supervision and mentorship of health workers. Training health workers on COVID-19 prevention and management and other services. Communicate guidance on the maintenance of health services and other guidelines. Mobile applications facilitated adherence to the MOH COVID-19 guidance by community health workers. Phones supported improved communication between community health workers and health facilities.
Maintenance of essential health services	Communication campaigns about adjustments to critical services delivery and encourage continued access to health services. Provision of information about services, conduct triage and appropriately refer clients. Reach out to clients with scheduled clinic visits such as through phone calls. Electronic logistics management information system facilitated the procurement process for COVID-19 commodities. Provision of health services including health information remotely including consultations, mental health and psychosocial support, and laboratory testing. Ordering and delivery of health products and commodities. Providing instructions such as through videos and follow-up of clients to ensure medication adherence and reduce lost to follow-up. Reach out to district or health authorities to avail transport for critical services such as delivery. Establishment of toll-free telephone numbers for victims of gender-based violence to access services. Training youths to use social media platforms to advocate for services utilisation, such as contraceptives

MOH, Ministry of Health.

### Testing

The response to the pandemic required rapid access to testing services and reduced turnaround time to ensure results that would inform isolation, quarantine, contact tracing or discharge among others. COVID-19 testing was initially centralised in the public sector, but as demand for laboratory testing services increased, the private sector was involved. At the peak of the COVID-19 waves when community transmission was evident, the MOH working with partners quickly acquired and deployed mobile laboratory vans fully equipped with COVID-19 testing capacities to ease access to testing and reduce turnaround time. For proper dispatch and management of COVID-19 test results, the MOH leveraged the centralised electronic results download system (eRDS) controlled at the district level that had been used for HIV/Tuberclosis (TB) care. Selected officers at the district health office and hospitals were given access rights which enabled instant access to laboratory results to support quick public health action. A short message service (SMS) was sent to individuals who had negative results while COVID-19-positive clients were called over the phone and followed up in-person. At the airport, LabXpert, a Laboratory Information Management System (LIMS) that manages the entire testing process ensuring patient information accessibility, accuracy, timeliness, security and confidentiality, was installed and integrated with the national result dispatch system (RDS). To operationalise the mandatory COVID-19 testing at Entebbe International Airport, the country used SMS and email to share results with incoming travellers and had a system to support registration and payment for the test before arrival to ease screening. The eRDS facilitated communication of test results from laboratories and could produce print reports accessible by key stakeholders in the laboratory or clinic and results channelled through the emergency operations centre to facilitate response. When some complaints were raised concerning discrepancies in laboratory results and forgery of COVID-19 test results, the ministry teams together with private laboratories included a quick response code to support verification and traceability of results. When rapid diagnostic testing kits were introduced, the MOH instituted two systems for healthcare workers to send data from facilities to the central level: the SMS-based reporting tool called mTrac and an internet-based reporting system called eLIF (electronic lab investigation form) with a direct data pipeline to the electronic results dispatch system (eRDS). As manual COVID-19 certificates and delayed test results caused extensive delays at border points, the East African member states developed the Regional Electronic Cargo and Driver Tracking System (RECDTS). RECDTS was a mobile phone application which enabled the issuance of the COVID-19 digital certificates that were mutually recognised by member states eliminating the need for multiple testing and easing congestion at East Africa’s border crossing points.

### Contact tracing and surveillance

Digital health tools and information systems played a critical role in COVID-19 contact tracing and surveillance. At the beginning of the pandemic, the MOH put in place call centres with toll-free lines to manage alerts from the community and support case finding and contact tracing. Go.Data, a free contact tracing tool by the WHO was extensively used for contact tracing, especially at a point where manual contact tracing was no longer feasible due to increasing COVID-19 cases in Uganda. Go.Data collected case data from laboratories, hospitalisation records and contact tracing. At the districts, the surveillance officer received data through phone calls, SMS or WhatsApp from health workers, community health workers (CHWs) and community members, and used it to compile reports which were shared with the MOH. A Virtual Emergency Operations Center mailing platform (VEOCi) was also created to support sharing of daily situational reports of the district subcommittees with the MOH. The Electronic Integrated Disease Surveillance and Response (eIDSR) system, built on the District Health Information Software 2 (DHIS2) platform, also collected COVID-19-related data to support surveillance activities. Data collected through the eIDSR system included COVID-19 contacts, results of laboratory investigation and tests conducted, confirmed cases and their management. A Point of Entry Data capture module was later integrated into the eIDSR platform to support screening, clearance and tracking of travellers at border districts. The Open Data Kit (ODK) forms, created and uploaded on mobile phones or computers, collated alerts, calls and cases which also supported surveillance activities at the district and national levels. These technologies made it possible for the country to collect COVID-19 data which supported contact tracing, reporting, monitoring and surveillance of the disease.

### Risk communication and community mobilisation

During the response to the pandemic, the MOH developed multilingual information and communication campaigns to raise awareness and promote adherence to prevention measures. The ‘tonsemberera’ campaign which means ‘do not come near me’ was a multilingual information campaign developed to encourage social distancing to curb transmission of the virus from person to person and was broadcast through social and mass media platforms. The risk communication messages emphasised social distancing, avoiding crowds, wearing masks and washing hands using soap and water or sanitising gel. Some of these messages were used as caller tunes by telecommunication companies. Media houses worked jointly to ensure consistent messages such as testimonies of people who recovered during the first wave of COVID-19. The use of media facilitated the propagation of these messages.

*You know, at first people were not believing that COVID-19 was real, so we had a campaign with UNICEF on ‘COVID-19 is real’. We had testimonies from the people relayed on different media platforms which helped us to really communicate the risk.* (KI, MOH)

U-Report, a social messaging tool and data collection system developed by UNICEF to improve citizen engagement, inform leaders and foster positive change, was used to share COVID-19 information and messages through mobile phones. The UN Innovations Data Lab used machine learning with a text-to-speech radio monitoring technology to identify incidents, beliefs and opinions about the pandemic which guided risk communication messaging. Reports of infections and strain on healthcare systems supported healthcare professionals in early investigation, response and resource prioritisation. These platforms also supported community engagement, combatting misinformation, responding to community questions and concerns, and gauging the pandemic’s social and economic impacts.

### Supportive supervision and training

Digital technology was also applied in supportive supervision, mentorship and training of health workers. Electronic platforms such as Zoom and WhatsApp were used to communicate guidance on the maintenance of health services and train health workers on COVID-19 prevention and management^26^ and supervision and coordination of activities. Some implementing partners transitioned from in-person training to online training, for example, in the provision of family planning services. The MOH in partnership with Living Goods, a private company, also developed a mobile phone application that CHWs uploaded on their smartphones to facilitate adherence to the ministry’s guidance on preventing COVID-19 as they provided care and treatment for malaria, diarrhoea and pneumonia for children under 5 years, as well as supporting mothers with antenatal and postnatal care.^27^ Other civil society organisations provided smartphones to CHWs to improve communication with health facilities and facilitate community health work. A local consortium working with the MOH set up a call centre with a team of nurses and doctors to provide a network of 3500 CHWs in 23 rural districts with COVID-19 information and support them in identifying, referring and managing COVID-19 cases.^28^ The consortium provided CHWs with a phone and tablet and met frequently for further training and support supervision.

### Maintenance of essential health services

The MOH used technology for communication campaigns encouraging people to continue accessing health services while protecting themselves from COVID-19. The MOH used traditional and social media platforms to communicate to the public about adjustments to critical services delivery and the provision of specific services at designated facilities and encouraged facilities to call clients with scheduled clinic visits. The MOH AIDS Control Programme promoted HIV testing through the development and dissemination of HIV self-testing videos in multiple languages. The use of technology was expanded in other areas including counselling over the phone, sending SMS texts to adolescents reminding them about clinic appointment dates, provision of family planning commodities using community-based condom dispensers, and training youth peers to use social media platforms to advocate for contraceptive use among adolescent girls and young women. Private health facilities and some public facilities provided the option of accessing healthcare remotely such as for consultations, mental health and psychosocial support, and provision of health information.^20^ These services were provided through voice, chat and video platforms and SMS.^20^ Some private delivery companies also provided options for ordering and delivering health products and commodities as well as sample pickup for COVID-19 and non-COVID-19-related medical services through their online platforms, especially in the capital city and other urban areas. Beyond supporting access to services, technology was also used in the follow-up of clients. For example, to ensure medication adherence and reduce loss to follow-up, service providers called clients over the phone, sent SMS, or used video to support directly observed treatments. In some districts, phone numbers of health authorities were provided to the community to request transport and ease access to delivery services to reduce unsafe home deliveries. Call centres were also set up to provide information about services, conduct triage and appropriately refer clients.^20^ CHWs in some areas were also provided with mobile phones and tablets to support usual tasks, coordinate the supply of essential medicines and commodities, and receive routine guidance as they continued with services delivery. There was also the establishment of toll-free telephone numbers for victims of gender-based violence to access services.

*We resorted to making follow-ups using telephones, we bought quite a number of phones for calling clients with blood pressure cases so that they may not get lost, but also to make some follow-up on the telephone.* (KI, General Hospital)

## Challenges and gaps in digital technology use during COVID-19 response in Uganda

While Uganda quickly adapted to the use of digital technology to control COVID-19, this did not come without limitations. The limitations included multiple disparate digital tools and information systems; limited access and coverage of the tools, poor data quality; inaccessibility of data; inability to support data manipulation, analysis and visualisation; and poor internet and electricity infrastructure.

### Disparate digital tools and health information systems

Several digital technologies developed and adapted for different aspects of the COVID-19 response led to the existence of multiple digital tools and technologies that were disparate and in isolation from each other. These systems were not integrated with the National Health Information Management System (DHIS2), limiting data collation and real-time reporting. The country lacked a central system for collecting, analysing, interpreting and disseminating data. For instance, the districts used ODK-data capture forms and eiSDR to report, the Call Centre used a COVID-19 survey system to capture COVID-19-related alerts, and case reporting was done manually using WhatsApp, while reporting from the laboratory was also based on RDS and eLIF. As a result, several systems had to be consulted for comprehensive information on the progression of the pandemic, which was tedious and led to several challenges such as a heavy workload, incomplete and duplicate information, and delay in communication and decision-making. There was also frequent change in data collection tools and data demands as the pandemic trajectory evolved and this caused further confusion on tools to be used for reporting. The systems lacked interoperability, which limited the availability of real-time information for quick decision-making.

*…. the challenge is they are multiple systems for COVID-19 reporting. So, for the cases reported, we have the results dispatch, another system for reporting for the laboratory system… and then for the surveillance system, the strategic information pillar is in the process of developing a database on the DHIS2 platform where you can have detailed information about the cases and then the district reports using ODK.* (KI, MOH)

### Limited access to and coverage of digital tools

With the development of several digital tools, access to some of them was limited by geographical barriers especially the limited access to technology infrastructure, electricity and internet which impacted data collection, access to essential health services, and training and supervision of health workers. There was also a lack of skills to use the instituted tools in some health facilities and regions which also extended to CHWs and facility clients impacting efficiency. Beyond skills, language was an issue especially in access to information by the community and provision of remote services including consultations and counselling.

### Poor data quality

The other major issue was the poor quality of data received which often contained errors and was incomplete. Rapid diagnostic tests (RDTs) were distributed to some health facilities that were not linked to the eRDS system, making real-time reporting a challenge. The investigation forms were complex and labour-intensive and the submitted data was often incomplete. As a result, teams utilising the data were required to clean and validate it before analysis. In some departments, extra people were recruited to support data cleaning. A team of duty officers were called on weekly to audit and verify the data before utilisation. This delayed availability of real-time data for decision-making. Gaps also existed in reporting services accessed through telemedicine via the usual health management information system.

*You have to subject the data to another level of cleaning, sometimes it is poor quality data, so it has to be cleaned and analysed separately before you present the summaries to any decision maker.* (KI, MOH)

Sometimes the data systems broke down and this led to manual data entry which increased errors in the data. For example, the existing platforms for laboratory surveillance information systems were overwhelmed by the heavy workload and they frequently broke down.

*This one [LIMS] caused us a glitch and we started having results manually entered into the system and because of those manual entries, we would have errors.* (KI, MoH).

### Inaccessibility of data

To ensure quick access to COVID-19-related data, several partners collaborated with the MOH to implement systems that supported reporting, such as ODK. These systems, however, were managed by funders and implementing partners hence access to the information by key stakeholders including the MOH was not always guaranteed. As a result, some data were not accessible and could not be used for decision-making. Various organisations also required districts to submit daily reports using different reporting templates which increased the workload.

*We had a challenge of coordination in national systems and the different partners who were supporting those systems…. because of that, I am afraid that some of the data were in the hands of implementing partners and we did not access it and may probably not be easily accessed by national systems.* (KI - MOH)

### Inability to support data manipulation, analysis and visualisation

While some systems had dashboards for data visualisation, most of the available systems lacked data aggregation and analytical capabilities. This made the process of data analysis tedious and manual. Data had to be manually extracted from each of the systems and then input into external software such as Microsoft Excel for cleaning, analysis and visualisation. Both the VEOCi and WhatsApp Platforms lacked data aggregation and analysis for real-time reporting.

*Data analysis was still mostly manual as our systems do not have that automation that allows you to visualise data in form of either graphs, maps or tables in real-time. So, you still have to extract the data and do an analysis separately outside the main system… we had to make a deliberate effort to analyse and make meaning of the data*. (KI - EOC)

### Poor internet and electricity infrastructure

Limitations in access to electricity and the internet in some areas hindered coverage of communication campaigns regarding availability of essential health services and that of health services offered remotely including their timeliness and quality. The poor infrastructure also delayed timely data submission from communities, health facilities and the districts to the centre. This led to slow updating of data for some districts. Areas with poor internet connectivity resorted to the use of manual or alternative tools like WhatsApp which increased the required workload to collate and synthesise data. The cost of the internet was also prohibitive for some facilities impacting frequency and timeliness of reporting, access to trainings and follow-up of clients. By June 2020, only 56% of the districts were successfully submitting daily reports due to limitations such as a lack of internet and poor network connections in some areas.

## Conclusion

In Uganda, digital technology supported several response activities including testing, contact tracing and surveillance, risk communication, training activities and maintenance of essential health services. The challenges with digital technology use were the disparate digital tools and health information systems leading to duplication of effort; limited access and coverage of digital tools, poor data quality; inaccessibility of data; and inability to support data manipulation, analysis and visualisation. The inadequate technology support systems such as poor internet/electricity infrastructure were also reported.

During the COVID-19 pandemic, Uganda quickly adapted existing technologies and created new ones to combat the disease based on its experience in responding to other public health threats. Stakeholders reported that technology increased the efficiency and accuracy of various response activities and the availability of real-time data for decision-making such as during testing, contact tracing and surveillance. Digital technology also supported risk communication and community mobilisation and training health workers. With the increasing access to mobile phones and the share of those who rely on the internet for information, the MOH used social media sites and mass media to share health information and deal with misinformation. The use of social media for health communication continues to gain traction^20^ including among health workers^29^ and should be further harnessed. One area for improvement is to diversify messages to other local languages to increase their effectiveness among the local population. Another role of technology that should be enhanced is its use in training health workers which reduced the potential of exposure to infection and minimised time and access barriers. Virtual reality simulations for training front-line health workers are feasible and can achieve better outcomes in skills acquired, speed of learning and rates of information retention compared with classroom training.^30^ Further evaluations of virtual training options are required to learn the modalities of how it can be effectively applied for health worker mentorship, support supervision and training. Digital technology also supported the maintenance of essential health services. As the pandemic also came with restrictions related to physical contact, telemedicine provided the opportunity to access health services without increasing the risk of COVID-19 infection. Indeed, the use of teleconsultation, telepsychiatry, call centres and information dissemination through mobile phones in Uganda increased.^25^ In sub-Saharan Africa, telemedicine use during the pandemic offered the opportunity to continue medical services provision and provide education and mental health and psychosocial support to clients.^20^ However, gaps in regulatory frameworks and policies, digital barriers, funding, technologies and biases among healthcare workers were reported.^15 16 31^ In Uganda, geographical limitations hindered access to on-demand health services as these were mostly in the capital city. Internet charges and language barriers also hampered the use of teleconsulting and access to information by the population. Health worker capacity was also limited.^20^ Among CHWs who had been supported through telehealth in rural Uganda, the use of the established call centre was suboptimal.^28^ These gaps should be bridged to accelerate the inevitable adoption of telemedicine in Africa to address current and future gaps in access to healthcare including during epidemics.

The application of digital technology in dealing with the fast-evolving pandemic came with some challenges. The emergence of multiple unregulated electronic tools, a challenge that existed and was exacerbated during COVID-19, limits their utility and sustainability, and may also pose challenges and risks to the community. Coordination of the various partners including development partners, the private sector and researchers who introduced other technology systems not aligned or integrated with the MOH platforms was a challenge which led to limited access and use of the collected data. The duplication of technology tools increased the tedium of data collection and collation reducing effectiveness and contributing to poor data quality. Also, many of the digital technology tools in the COVID-19 response were not designed to support easy data manipulation, analysis and visualisation. Using technologies during the pandemic led to a neglect of data protection and a lack of a clear framework for data sharing among stakeholders. These challenges point to broader data governance challenges within the health system which come with data ownership, access, security issues, privacy and confidentiality concerns as acknowledged in Uganda’s Health information and digital health strategic plan 2020/2021–2024/2025.^32^ A recent mapping revealed that between 2010 and 2020, Uganda’s health system used 91 digital health tools, 35 of which had been deployed to support the COVID-19 response. Prior to future epidemics, technologies should be developed, piloted, adopted and streamlined to ease their adaptation and use during crisis. The MOH should devise a clear policy to guide technology adoption during epidemics which should have a coordination mechanism for all stakeholders and emphasise integration with pre-existing systems and full access to data by the ministry. The MOH should harmonise all surveillance and reporting tools and technologies, integrate appropriate tools and ensure their interoperability for information exchange to improve data reporting at the local level and real-time decision-making to increase impact. MOH is also better placed to guide on the priority gaps to ensure that innovations are responsive to their needs and build on existing systems. The policy should also clearly stipulate measures to ensure data protection for clients, patients, communities and citizens. Broadly, there is need for a systematic review of the tools that were introduced during COVID-19 to identify those that are useful and scalable, and document lessons to enhance policy and regulatory environment. In the adoption and use of technology, utmost steps should be taken to ensure that they do not increase inequity gaps in healthcare access and outcomes. Indeed, bolstering and improving internet and electricity infrastructure, the use of low-cost technological options, and increasing computer skills and access to equipment and technologies are important for wider adoption of digital health technologies in low-income and middle-income countries and to bridge inequity gaps.

This study draws from the policy-makers’ and health workers’ perspectives to highlight the application of digital technology in the COVID-19 response in Uganda. Future studies could consider exploring user perspectives around these technologies which would provide a broader perspective and inform more lessons for the future. It would also be important to examine the effectiveness of some of the technological interventions and their contribution to the pandemic response. Equity in the availability and utilisation of digital tools across different regions, districts, facility levels and population groups also requires further examination.

Digital technology is increasingly important for bridging health systems gaps and proved instrumental to enhancing various components of the COVID-19 response in Uganda including testing, contact tracing and surveillance, risk communication, and training activities, and maintenance of essential health services. Several challenges, however, impacted the use of digital technology including the disparate digital tools and health information systems; limited access and coverage of digital tools, poor data quality; inaccessibility of data; inability to support data manipulation, analysis, and data visualisation; and poor internet and electricity infrastructure. There is a need for a clear policy and regulatory framework to guide technology adoption, evaluation, harmonisation, scalability and sustainability as well as coordination of various stakeholders during epidemics.

## Data Availability

Data are available on reasonable request.
