# Supplementary material for: Harnessing digital technology for COVID-19 response in Uganda: lessons and implications for future public health emergencies
Source: BMJ Glob Health. 2023 Oct 4;8(Suppl 6):e013288. doi: 10.1136/bmjgh-2023-013288 (PMC10551983; doi:10.1136/bmjgh-2023-013288)
Supplement: Supplementary data [file bmjgh-2023-013288supp001.pdf]

Harnessing digital technology for COVID-19 response in Uganda: Lessons and implications for future public health emergencies

Identifiers

a) Institution:
b) Office/Position:
c) Duration in the position:
d) Profession:
e) Role in the covid-19 response:

Digital technology use

1. Which digital technology systems were used to support aspects of the COVID-19 response in Uganda and how were they used? (Probes: At what level: health facility or community? What was the coverage of the technologies? Were these tools already existing or were they developed specifically for the COVID-19 response?)
   a) Screening and testing
   b) Surveillance and contact tracing
   c) Health workers support activities such as training and supervision
   d) Community mobilisation
   e) Maintenance of essential health services
   f) Reporting cases or sharing information
   g) Any other COVID-19-related activities.

2. How was data from digital technologies used to inform interventions? How was it disseminated? What key decisions did this data inform?
3. What were the facilitators of the use of digital technologies in COVID-19 response?
4. What challenges were experienced in the use of digital technologies for the various COVID-19 response functions? (Probe: who reported the challenges? how were the challenges resolved?
5. What gaps existed in the deployment and use of digital technologies during the COVID-19 pandemic?
6. Comment about the equity issues that arose in the use of digital technologies during the COVID-19 response in Uganda?
7. What recommendations do you have to improve the deployment and use of digital technologies during pandemics in Uganda?
